# The Usefulness of Contrast-Enhanced Ultrasound in the Assessment of Early Kidney Transplant Function and Complications

**DOI:** 10.3390/diagnostics7030053

**Published:** 2017-09-15

**Authors:** Sara Álvarez Rodríguez, Vital Hevia Palacios, Enrique Sanz Mayayo, Victoria Gómez Dos Santos, Víctor Díez Nicolás, María Dolores Sánchez Gallego, Javier Lorca Álvaro, Francisco Javier Burgos Revilla

**Affiliations:** Kidney Transplant Unit, Department of Urology, Hospital Universitario Ramón y Cajal, Spain Alcalá University, IRYCIS, 28034 Madrid, Spain; vital.hevia.uro@gmail.com (V.H.P.); esm_75@hotmail.com (E.S.M.); vgomezd69@gmail.com (V.G.D.S.); victordnicolas@gmail.com (V.D.N.); lolasaga11@gmail.com (M.D.S.G.); javier.lorca.alvaro@gmail.com (J.L.Á.); burgoss2000@yahoo.es (F.J.B.R.)

**Keywords:** CEUS, contrast enhaced ultrasound, kindey transplant, complications

## Abstract

Objectives: The routine diagnostic method for assessment of renal graft dysfunction is Doppler ultrasound. However, contrast-enhanced ultrasound (CEUS) may provide more information about parenchymal flow and vascular status of kidney allografts. The aim of the study was to assess the effectiveness of CEUS in the immediate post-transplant period, focusing on acute vascular complications. A brief review of available literature and a report of our initial experience is made. Material and methods: 15 kidney transplant (KT) cases with clinical suspicion of acute surgical complication were assessed with CEUS and conventional Doppler ultrasound (US). In addition, bibliographic review was conducted through PubMed, Embase, and ClinicalKey databases. Results: 10% of KT underwent CEUS, useful for detecting vascular complication or cortical necrosis in 4 (26%) and exclude them in 74%. Grafts with acute vascular complications have a delayed contrast-enhancement with peak intensity lower than normal kidneys. Perfusion defects can be clearly observed and the imaging of cortical necrosis is pathognomonic. Conclusions: CEUS is a useful tool in the characterization of renal graft dysfunction with special interest on acute vascular complications after renal transplant. It is a feasible technique for quantitative analysis of kidney perfusion, which provides information on renal tissue microcirculation and regional parenchymal flow. Exploration could be done by a urologist at the patient’s bedside while avoiding iodinated contrast.

## 1. Introduction

Despite advances in transplantation and allografts preservation, complications after KT are unavoidable. Visualization of parenchymal properties and vascularization patterns of transplanted kidneys are key elements in the follow-up during the immediate post-KT period. Complications at this period include vascular (e.g., arterial or venous thrombosis, arterial stenosis, leaks or pseudoaneurisms, and cortical necrosis), urological or surgical complications (e.g., urinary leaks, lymphocele, urinary tract obstruction, bleeding, and hematoma), and infectious (e.g., renal abscess and pyelonephritis) and nephrological causes (e.g., acute rejection (AR), acute tubular necrosis (ATN), and anticalcineurin toxicity) [1].

Early diagnosis and treatment of these complications are important because immediate resolution of them has an impact on graft survival and long-term function.

Conventional Doppler ultrasound (US) has limited accuracy in the identification of major causes of graft dysfunction during immediate post-KT [2]. It provides information about transplanted kidney morphology and perfusion. However, it has limitations providing information regarding microcirculation, such as resistive index (RI) or pulsatility index (PI), as they are measured in interlobar and arcuate arteries and do not reliably reflect what happens distally at microvessels [3].

CEUS has several advantages compared to other commonly used techniques. It avoids nephrotoxicity and provides a reliable method for graft monitoring in the immediate post-transplant period, allowing a quantitative analysis of renal microvascularization, especially at sub-capsular vessels. It provides crucial information about several conditions that may lead to graft dysfunction in the immediate post-KT period [4].

In this article, we review the current state of this technique in the setting of KT, focusing on acute complications during the immediate post-KT period.

## 2. Material and Methods

**Figure 1:**
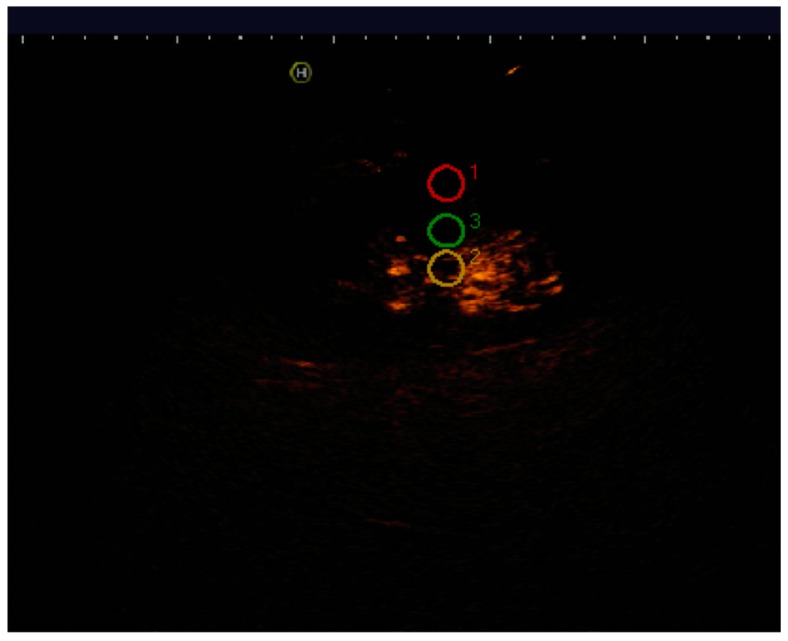
Placement of the three circular regions of interest (ROIs) in medullary region, interlobar arteries, and subcapsular cortex for the analysis of intensity curves (TIC).

**Figure 2:**
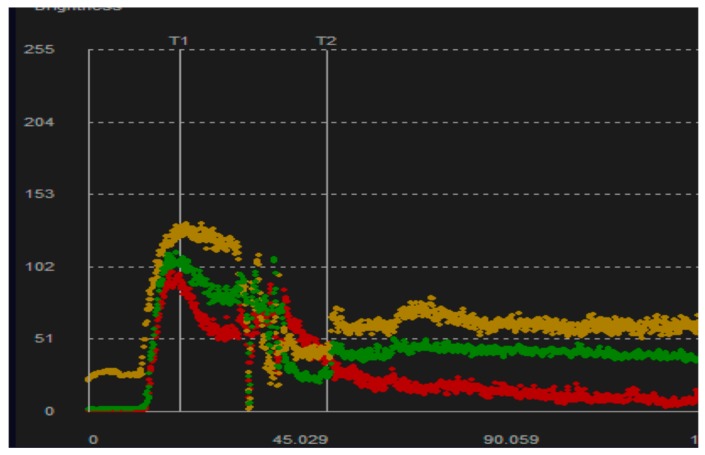
TIC after contrast injection showing the contrast-enhancement and washout at the three ROIs.

One hundred forty-seven patients were transplanted at our Institution in a two-year period between January 2015 and December 2016. A total of 15 patients (10.2%) underwent both CEUS and conventional Doppler ultrasound, 2–4 days after surgery. Indications for CEUS were clinical suspicion of vascular complications, unexplained initial graft dysfunction, or post-surgical control after complex surgery. After injection of 2.5 cubic centimeter (cc) of intravenous contrast agent (Sonovue^®^), intensity curves (TIC) were assessed placing three circular regions of interest (ROIs) in the medullary region, interlobar arteries, and subcapsular cortex (Figure 1). Initial baseline analysis was performed, and TIC parameters were correlated with clinical data and results from computed tomography (CT) and gammagraphy if available. In the case of biopsy or transplantectomy, pathological specimens were also analyzed. Analyzed parameters on CEUS were as follows: arrival time (AT) (time to injection to start of micro bubble uptake in seconds); time to peak (TTP) of maximum value; intensity peak of enhancement (IP); and final intensity (FI). Difference between IP and FI was calculated to evaluate the slope of the intensity curve (Figure 2). Parameters were always measured in the three ROIs described. Macroscopic visual evaluation of renal parenchyma was also considered.

Arterial flow is determined in the interlobar arteries and in the renal cortex using TIC. The difference in peak time intensity between these two vascular territories is determined, and the perfusion gradient is defined as the rate of increase of TIC from the interlobar artery to the renal cortex.

The initial contrast agent display occurs between 15 and 25 s and the optimal uptake is between 35 and 45 s. For quantitative analysis of renal perfusion, a commercial software tool is used, and an area of interest is selected at the renal subcapsular cortical level, the interlobar and arcuate arteries [5].

A bibliographic review was performed through PubMed, Embase, and Clinical Key databases, with a time restriction to papers published over the last 10 years regarding the use of ultrasound contrasts. Search criteria used were “contrast enhanced ultrasound”, “kidney”, “renal transplant”, and “kidney transplant”. The database search was complemented by screening the reference list in the included studies.

## 3. Results

**Figure 3:**
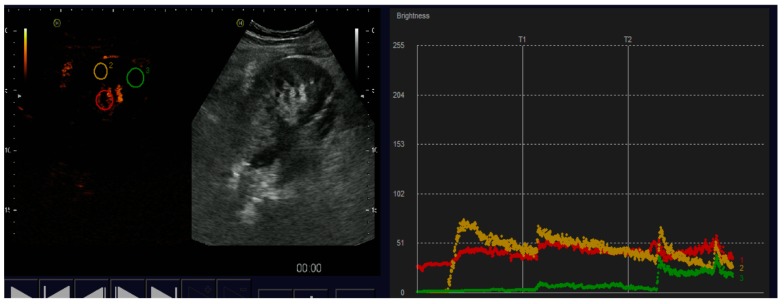
The slope in grafts with acute complication is flattened identifying a worse washout of contrast.

**Figure 4:**
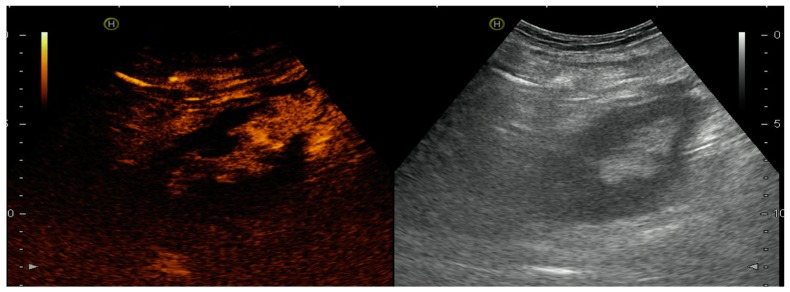
Partial absence of contrast-enhancement at the upper pole showing a focal ischemia secondary to a polar artery thrombosis.

**Figure 5:**
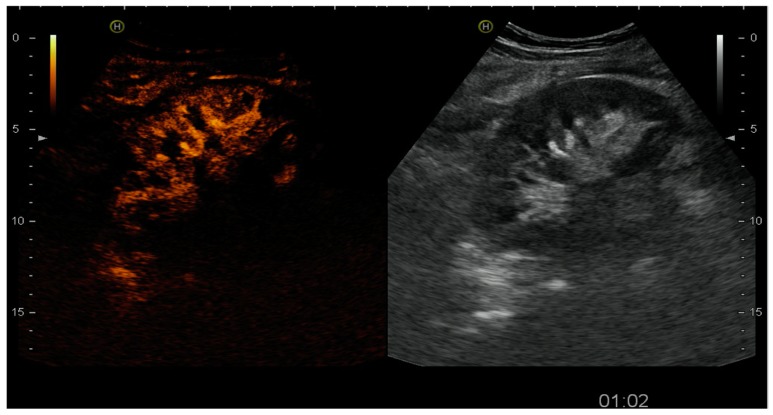
Cortical necrosis with an anechoic cortical halo.

**Figure 6:**
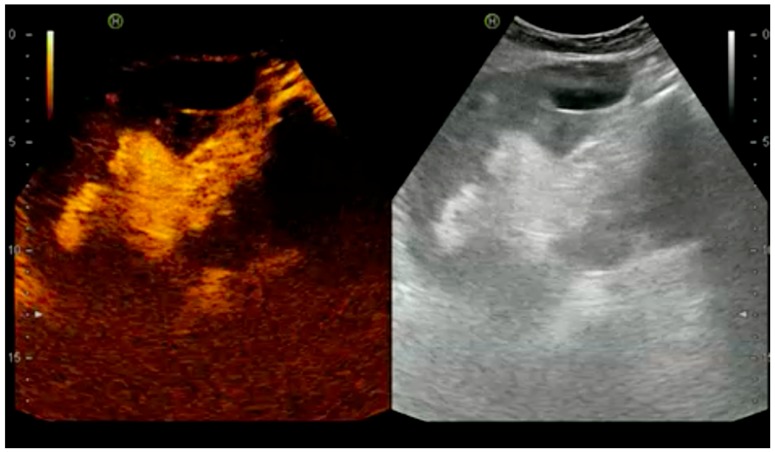
Renal abscess at the lower pole, seen as anechoic areas without contrast-enhancement.

Fifteen KT recipients were included: 13 from deceased donors (12 heterotopic in iliac fossa and 1 orthotopic), 1 living donor, and 1 auto-transplant. CEUS was useful to detect vascular complication or cortical necrosis in 4 (26%) and exclude them in 74%. Two patients showed reduced intensity curves, suggesting that ATN was the cause of the delayed graft function (DGF) (Figure 3). One infectious abscess was observed as an anechoic parenchymal area without contrast-enhancement (Figure 4). Two cortical necrosis presented a typical anechoic cortical halo (Figure 5), which cannot be detected by conventional US, and were confirmed with CT. One patient had a renal vein thrombosis, and one patient a focal ischemic area due to polar artery thrombosis (Figure 6). Ischemic areas appeared as a perfusion defect without contrast-enhancement. Patients with cortical necrosis and venous thrombosis underwent graft nephrectomy, and pathological analysis confirmed CEUS findings. The slope in grafts without evidence of acute complication was steeper identifying a better washout of contrast.

## 4. Discussion

Echo-enhancer contrasts are protein compounds, saccharides, or lipids with a central core filling gas that, when administered intravenously, have the ability to enhance the ultrasound signal [6]. They are formed by microbubbles smaller than 7 μm in diameter and thus can pass through the pulmonary circulation where they are eliminated avoiding renal excretion. The most commonly used contrast nowadays are Levovist^®^ (Schering, Berlin, Germany) and Sonovue^®^ (Bracco, Milan, Italy). Microbubbles in the tissue are destroyed by the high-energy ultrasound waves and provide information about flow and tissue perfusion.

Its main advantage compared to contrasts used in CT or MRI is that they do not spread outside the vascular space, so they are non-nephrotoxic and can be safely used even in patients with renal failure [7]. Their only contraindication is severe respiratory failure.

The interface between the sulfur hexafluoride microbubbles and the aqueous medium acts as a reflector for the ultrasonic wave by improving blood echogenicity and increasing the contrast between the blood and surrounding tissue. The reflectivity depends on microbubbles concentration and the frequency of the ultrasonic wave. A second injection may be repeated when the examining physician deems necessary. Kidneys are rapidly and intensely enhanced due to the high renal perfusion; renal arteries are enhanced in 10–15 s, renal cortex is enhanced 5–10 s later, and pyramids are enhanced afterward (30–40 s after initial). Finally, contrast is eliminated and the image goes from hyper- to hypoechoic progressively. The collecting system is not modified, as the contrast is not excreted by the kidney [3].

In grafts with acute vascular complications, contrast-enhancement is delayed and the peak intensity is lower than in normal kidneys [8]. Lebkowska [9] assessed graft perfusion after KT with CEUS; a correlation was observed between the glomerular filtration rate (eGFR) and blood flow parameters within the renal arteries, as well as between the flow time of contrast medium from the artery within the renal hilum to the cortex.

### 4.1. Surgical Complications

CEUS has become relevant to diagnose surgical complications during the immediate post-transplant period. It is a quick bedside test not influenced by immunosuppressive drugs and avoids nephrotoxicity. Moreover, it can be easily performed by transplant teams with experience in US, including urologists.

#### 4.1.1. Arterial Thrombosis

Arterial thrombosis incidence ranges between 0.9% and 2.5% [10], and its early diagnosis is a unique opportunity for intervention to save kidney viability. In case of complete arterial thrombosis, there is a complete absence of renal enhancement and an absence of venous flow. In the case of multiple pedicles, a focal ischemic area without contrast perfusion can be observed [10], as shown in Figure 4. In this case, surgical revision confirmed a lower pole infarction and allowed the resection of the ischemic segment and graft preservation.

#### 4.1.2. Venous Thrombosis

Venous thrombosis occurs with a slightly lower incidence than arterial thrombosis (0.6–2%). Absence of vein enhancement and reverse diastolic flow within the renal artery are the most common findings. Renal enhancement in the first few seconds of the cortical phase is pulsatile rather than continuous, probably due to congestion of the graft resulting in increased resistance to arterial flow, and cortical patches of hypocaptation can be seen. At this point, trasplantectomy is mandatory.

#### 4.1.3. Acute Cortical Necrosis

An infrequent but serious vascular complication with irreversible graft dysfunction is acute cortical necrosis. The pathogenesis still remains unclear and is often multifactorial: vasospasm, microvascular injury, and disseminated intravascular coagulation. Kidney damage is permanent and functional loss is irreversible. In our series, CEUS detected an anechoic cortical halo in two cases, and findings were overlapping to the peripheral rim sign in CT, which is pathognomonic of cortical necrosis. Trasplantectomy was performed and anatomopathological analysis confirmed diagnosis. In a retrospective study of five patients with pathology-proven acute cortex necrosis, only CEUS imaging but not conventional US was able to diagnose acute cortex necrosis [11].

#### 4.1.4. Infectious Complications

Complicated bacterial pyelonephritis may produce focal pyelonephritis, which may become an abscess. The usefulness of contrast in this situation is to detect and define the extent of pyelonephritis areas. Abscess is seen as anechoic areas without contrast-enhancement.

Presence of ischemic parenchymal lesions seems as triangular hypoechoic, hypoperfused areas of medulla at the CEUS are seen at acute pyelonephritis. Areas of hypoperfusion could also occur in acute rejection, but the hypoperfused areas are diffuse in that case. An anechoic area without contrast-enhancement at the lower pole was seen in our case, and surgical revision was performed, allowing local drainage and avoiding graft loss. CEUS has been compared to gadolinium MRI in recent studies for this purpose and seems to replace MRI for diagnosis of pyelonephritis [12].

#### 4.1.5. Arterial Stenosis (TRAS)

This is the most frequent vascular complication. Its incidence ranges from 1% to 23% and prevalence from 1.5% to 4% [13]. Conventional color Doppler imaging has shown to be the diagnostic mainstay for TRAS [14]. With CEUS, longer time of contrast agent inflow compared with patients without perfusion defects was observed and a contrast agent inflow was correlated with the severity of stenosis. Nevertheless, TRAS could be accurately diagnosed by conventional color Doppler imaging. No cases were found in our series.

#### 4.1.6. Perirenal Collections

Post KT collections are common findings that usually are asymptomatic and do not require additional interventions. Occasionally, they can cause vascular compression or urinary tract obstruction, requiring treatment. Hematoma, lymphocele, or urinoma are visualized as anechoic images without contrast-enhancement [15]. The benefits of CEUS are its permeability of vessels adjacent to collections, its ability to distinguish hematomas from perirenal tissue, and its improved extension definition.

### 4.2. Medical Complications

After KT, renal function is monitored usually by US. RI is non-specific, so additional measures should be assessed to verify microperfussion. Non-specific patterns have been described for DGF causes. In fact, US is usually used at this point to exclude surgical correctible causes or to guide biopsies.

At present, diagnosis of AR, apart from clinical suspicion, is commonly found in the study of vascular perfusion of the graft by determination of PI and RI by renal Doppler US [16]. RI is non-specific and may be influenced by multiple factors, including those unrelated to kidney disease, such as increased intra-abdominal pressure, pulse rate, treatment with calcineurin inhibitors, place of measurement, or high body mass index (BMI), as well as the presence of systemic diseases such as atherosclerosis, otherwise frequent in expanded criteria donors.

Cortical perfusion seems to decrease during acute antibody rejection [8], reflecting on TIC as a decrease of the area under the curve (ROC curves), which indicates a temporal difference in the initial signal between the renal cortex and the renal pyramid. This finding can also be seen in cases with DGF.

Some studies have shown a delay in cortical enhancement (based on renal artery enhancement) in patients with AR. This aspect, however, is not specific and can be observed also in patients with DGF and even in patients with large perirenal collections due to kidney or vessels compression.

## 5. Conclusions

The use of CEUS has been expanded in the study of cardiac, hepatic, and gynecological pathology. Its use in evaluation of KT is increasing both in the immediate and in the long-term setting with better visualization of macro- and microvascularization, improving Doppler results. CEUS is a useful tool in the characterization of renal graft perfusion. Special interest should be focused on the diagnosis of acute complications after renal transplant, especially vascular ones. In our experience, it allowed accurate diagnosis excluding surgical complications in 74% of cases and enabling quick resolution of any potentially treatable surgical or vascular complication in the immediate post-KT. It can be performed by transplantation teams at a patient’s bedside while avoiding the nephrotoxic effect of iodinated agents.
